# Identification of Cooperative Gene Regulation Among Transcription Factors, LncRNAs, and MicroRNAs in Diabetic Nephropathy Progression

**DOI:** 10.3389/fgene.2020.01008

**Published:** 2020-09-01

**Authors:** Ling Chen, Binbin Wu, Shaobin Wang, Yu Xiong, Boya Zhou, Xianyi Cheng, Tao Zhou, Ruibang Luo, Tak-Wah Lam, Bin Yan, Junhui Chen

**Affiliations:** ^1^Intervention and Cell Therapy Center, Peking University Shenzhen Hospital, Shenzhen Peking University-The Hong Kong University of Science and Technology Medical Center, Shenzhen, China; ^2^Shenzhen Engineering Laboratory of Nanomedicine and Nanoformulations, Institute of Biomedicine and Biotechnology, Shenzhen Institutes of Advanced Technology, Chinese Academy of Sciences, Shenzhen, China; ^3^Department of Ultrasound, The Eighth Affiliated Hospital, Sun Yat-sen University, Shenzhen, China; ^4^Department of Computer Science, Faculty of Engineering, The University of Hong Kong, Hong Kong, China

**Keywords:** diabetic nephropathy, transcription factors, long non-coding RNAs, microRNAs, regulatory interactions

## Abstract

The pathogenesis of diabetic nephropathy (DN) is accompanied by alterations in biological function and signaling pathways regulated through complex molecular mechanisms. A number of regulatory factors, including transcription factors (TFs) and non-coding RNAs (ncRNAs, including lncRNAs and miRNAs), have been implicated in DN; however, it is unclear how the interactions among these regulatory factors contribute to the development of DN pathogenesis. In this study, we developed a network-based analysis to decipher interplays between TFs and ncRNAs regulating progression of DN by combining omics data with regulatory factor-target information. To accomplish this, we identified differential expression programs of mRNAs and miRNAs during early DN (EDN) and established DN. We then uncovered putative interactive connections among miRNA–mRNA, lncRNA–miRNA, and lncRNA–mRNA implicated in transcriptional control. This led to the identification of two lncRNAs (MALAT1 and NEAT1) and the three TFs (NF-κB, NFE2L2, and PPARG) that likely cooperate with a set of miRNAs to modulate EDN and DN target genes. The results highlight how crosstalk among TFs, lncRNAs, and miRNAs regulate the expression of genes both transcriptionally and post-transcriptionally, and our findings provide new insights into the molecular basis and pathogenesis of progressive DN.

## Introduction

As a severe and frequent microvascular complication of diabetes, diabetic nephropathy (DN) is the leading cause of renal failure in developed countries. The prevalence of DN ranges from 20 to 40% in both type 1 and type 2 diabetic patients (Gheith et al., 2016), and it affects ∼50% of patients diagnosed with end-stage renal disease who need renal replacement (Kato and Natarajan, 2014). Current treatments of DN rely mainly on drugs that reduce its progression or on renal replacement therapies. As the number of diabetic patients has continued to increase over the last decades, DN has become a worldwide public health concern. During the pathogenesis of this disease, a clinically silent early DN (EDN) occurs prior to overt DN. EDN can either progress to overt DN or regress to normal and non-nephrotic conditions (Bjornstad et al., 2014). With the increased prevalence of EDN and need to prevent progression to DN, there is a need to identify both more sensitive and specific biomarkers for its early detection and molecular targets for improved clinical management. The identification of early molecular markers and targets would be important not only for understanding molecular mechanisms but also for providing clinical guidance to appropriately manage diabetic renal disease.

The pathogenesis of DN is associated with pathophysiological changes that involve a broad range of biological processes and signaling pathways (Pichler et al., 2017; Warren et al., 2019). These alterations occur concomitantly with changes in the expression of a large set of genes, but the initiation and progression of DN are likely controlled through the interactions of transcription factors (TFs), non-coding RNA (ncRNA), and other key regulatory mechanisms (Sanchez and Sharma, 2009). Nuclear factor kappa-light-chain-enhancer of activated B cells (NF-κB) is likely an important TF in the pathogenesis of DN, and it is activated by both metabolic and hemodynamic alterations caused by diabetes (Suryavanshi and Kulkarni, 2017). When activated, NF-κB is involved in the transcription and regulation of inflammatory cytokines, chemokines, cell adhesion molecules, and other molecules (Navarro-Gonzalez et al., 2011). Other TFs such as nuclear factor erythroid 2–related factor 2 (NRF2 or NFE2L2), peroxisome proliferator activated receptor gamma (PPARG), activator protein-1 (AP1), and hepatocyte nuclear factor-1-beta (HNF1B) are believed to control gene expression and mediate related functional alterations in DN (Kikkawa et al., 2003; Panchapakesan et al., 2009; Sanchez and Sharma, 2009; Ruiz et al., 2013; Clissold et al., 2015; Warren et al., 2019). However, the global effects of gene modulation caused by these TFs and other regulators in governing the progressive processes of DN has not been adequately defined.

Among ncRNAs, both microRNA (miRNA) and long ncRNA (lncRNA) have been reported to play disparate roles in diabetic diseases such as DN (Guo et al., 2019; Sankrityayan et al., 2019). Several miRNAs, like miR-27a, miR-29c, and miR-30a (Peng et al., 2015; Wu et al., 2016; Guo et al., 2017), may be involved in the progression of DN, and they are related to the development of inflammation, fibrosis, mesangial expansion, and podocyte injury. Let-7a-5p is down-regulated in mesangial cells under high glucose conditions and participates in the pathogenesis of DN by regulating phosphatidylinositol-3-kinase (PI3K) – protein kinase B (AKT) signaling pathways (Wang et al., 2019). miR-21 protects hyperglycemia-induced renal cells from apoptosis via inhibiting transforming growth factor beta (TGF-β) signaling and function (Lai et al., 2015; see Supplementary Table S1). Recently, an increasing number of studies have proposed lncRNAs being essential for the regulation of DN. Metastasis-associated lung adenocarcinoma transcript 1 (MALAT1), a widely expressed lncRNA, is involved in podocyte injury and reduces reactive oxygen species in endothelial cells (Liu et al., 2014; Hu et al., 2017). Taurine up-regulated 1 (TUG1), another lncRNA implicated in metabolic change, regulates mitochondrial function in podocytes via PPARG (Kato and Natarajan, 2014). Nuclear enriched abundant transcript 1 (NEAT1), which is up-regulated under diabetic condition, accelerates the proliferation and fibrosis in DN via activation of the AKT/mammalian target of rapamycin (mTOR) signaling pathway (Huang et al., 2019). Other lncRNAs like MIAT, PVT1, and H19 imprinted maternally expressed transcript are key mediators postulated to be involved in human DN (see Supplementary Table S2). Despite substantial efforts to link ncRNAs with DN, no direct interactions among lncRNAs, miRNAs, TFs, downstream genes, and the associated functional alterations have thus far been identified as essential for the progression of DN.

In this study, we analyzed multiple sets of EDN and DN mRNA and miRNA expression data to identify differentially expressed genes/mRNAs (DEGs) and miRNAs (DEMs). We developed a network-based system to identify interactive connections among the three types of RNAs. Our integrated approach led to the construction of gene regulatory networks of TF–lncRNA–miRNA–mRNA. These analyses led to the finding that cooperative regulation among TFs (NF-κB, PPARG, and NEF2L2), lncRNAs (MALAT1 and NEAT1), and a set of miRNAs plays a central role in the development and progression of DN. The results of our study improve understanding of the molecular basis of regulatory mechanisms underlying DN pathogenesis and provide potential targets for both monitoring and treating DN.

## Materials and Methods

### Data Source

We first extracted genome-wide microarray and RNA-seq data of mRNA and miRNA human from GEO ArrayExpress and reported in publications. The mRNA data include two sets for EDN versus (vs.) control (GSE111154 and GSE142025) and four sets for DN vs. control (GSE142025, GSE1009, GSE30528, and GSE96804). The GSE111154 dataset includes RNA expression profiles from eight blood and kidney tissue samples (four from EDN and four from non-diabetic control). The GSE142025 dataset includes RNA expression profiling of kidney biopsy samples from 28 patients with early and advanced DN and 9 patients as health control. The GSE1009 dataset includes gene expression profiles from six human kidney samples, which includes three patients with diabetes and three healthy controls. The GSE30528 dataset includes RNA expression profiling of human kidney samples from 9 diabetic patients and 13 healthy persons. The GSE96804 dataset includes gene expression profiles of micro-dissected glomeruli from diabetic patients.

The miRNA data used in this study consists of four sets for DN vs. control from GEO (GSE114477 and GSE51674) and E-MTAB-4166 and reported in Cardenas-Gonzalez et al. (2017). The GSE114477 dataset includes miRNA expression profiling of samples from 20 individual chronic kidney disease and 20 healthy controls. The GSE51674 dataset includes ncRNA expression profiles of kidney tissue from eight DN patients, six diabetic patients with membranous nephropathy, and four patients with normal histology. E-MTAB-4166 includes exosomal miRNA expression profiles of urine from eight DN patients, eight T2D patients, and eight healthy controls.

### Analysis of Differentially Expressed mRNAs and miRNAs

After the six normalized datasets described above were downloaded, we performed statistical analysis to identify DEGs. We used empirical Bayes moderated *t*-test that is provided in limma software under R environment. Fold changes of mRNA expression for the EDN or DN were calculated relative to control. DEGs were set at a cutoff of fold change at least 1.5 and *p*-value below 0.05. We used the reported miRNAs from the publications as DEMs.

### LncRNA-Target Analysis

Resources of lncRNA–mRNA or miRNA interactions included the following: (1) LncBases provide miRNA targets of lncRNAs, consisting of more than 70,000 low- and high-throughput, direct or indirect miRNA–lncRNA experimentally supported interactions (Paraskevopoulou et al., 2013, 2016); (2) NPInter databases^1^ provide interactions of ncRNAs including lncRNA–mRNAs and lncRNA–miRNAs, supported by experimentally validated and high-throughput experimental test (Hao et al., 2016; Teng et al., 2020); (3) LncTarD^2^ provides a comprehensive resource of key lncRNA-target regulations and lncRNA-mediated regulatory relations with human diseases (Zhao et al., 2020); and (4) starBase v2.0^3^ provides datasets of lncRNA–miRNA and mRNA–miRNA interactions based on CLIP-Seq experimental data (Li et al., 2014).

### miRNA-Target Analysis

There are two resources that can provide information for target mRNAs of miRNAs. First, mirTarBase is a database of miRNA target genes that is supported by experimental validation. Second, miRecords, a web-based platform, contains the predicted binding target genes of miRNAs based on 11 computational methods. Genes or mRNAs were considered as binding targets of a particular miRNA if they were identified by at least four methods.

We determined the similarity among expression patterns through co-expression analysis of miRNAs and mRNAs by calculating Pearson correlation coefficients (PCC). Problematically, the sample numbers differed among the experimental groups (DN, EDN, and control) for the miRNAs and mRNAs. We therefore assumed that all of the samples were random and representative between DN and EDN populations. To test this assumption, we should have a pairing of miRNA and mRNA. We have the PCC of miRNAs and mRNAs:

$$\rho \,x,y=\frac{\text{cov}\,\left(X,Y\right)}{\sigma_{X}\,\sigma_{Y}}$$

$$=r_{x\,y}=\frac{n\,\sum x_{i}\,y_{i}-\sum x_{i}\,\sum y_{i}}{\sqrt{n\,\sum x_{i}^{2}-{\left(\sum x_{i}\right)}^{2}}\,\sqrt{n\,\sum y_{i}^{2}-{\left(\sum y_{i}\right)}^{2}}}$$

where *x* and *y* denote the expression levels of miRNAs and mRNAs.

We can evaluate all possible pairings between miRNAs and mRNAs by permutation or a random sample of all possible combinations of samples and look at the distribution of possible *p*-values. Thus, we performed a permutation test for PCC by two steps:

1) To obtain a paired data (*x*_*i*_, *y*_*i*_), we permutated 1,000 runs by randomly selecting the same sample number “*i*” of DN or EDN and control for both miRNA and mRNA. The samples were randomly picked from their corresponding expression datasets based on the smallest number of samples.
2) After performing the runs 1,000 times, we calculated the possibility or how many coefficients of a miRNA–mRNA pair are significantly corrected under *p* <0.01. We selected the miRNA–mRNA pairs as true correlation relationships if satisfying the possibility of at least 95%.

### Functional Analysis

We used DAVID software to analyze the enrichment of Gene Ontology (GO) biological processes and Kyoto Encyclopedia of Genes and Genomes (KEGG) pathways among a set of genes.

### Transcription Factor Binding Site Analysis

To predict whether a gene is regulated by a TF, we identified TF binding sites conserved on human and mouse promoters. We searched for TF binding sites by using PWMSCAN (Wang et al., 2007). This method conducts computational identification of binding sites by scanning promoter sequences using position weight matrices of TF binding motifs. The predicted binding sites were evaluated by the *p*-value, which was calculated via the permutation-based method FastPval (Li et al., 2010). The putative binding sites were filtered based on conservation scores between human and mouse genomes. Promoters analyses were limited to locations 2,000 bp upstream and 500 bp downstream from the transcriptional start site.

### The Enrichment Analysis of Target Gene Sets

The statistical significance of enrichment level for the overlaps between target gene sets of different TFs or lncRNAs was assessed according to the hypergeometric probability distribution:

$$=\frac{\left(\frac{a}{k}\right)\,\left(\frac{N-a}{b-k}\right)}{\left(\frac{N}{b}\right)}.$$

where *k* = overlapped number of genes between the two gene sets, *N* = total gene number, *a* = gene number in gene set *A*, and *b* = gene number in gene set *B*.

## Results

### A Network-Based Pipeline to Build TF–lncRNA–miRNA–mRNA Network

**FIGURE 1:**
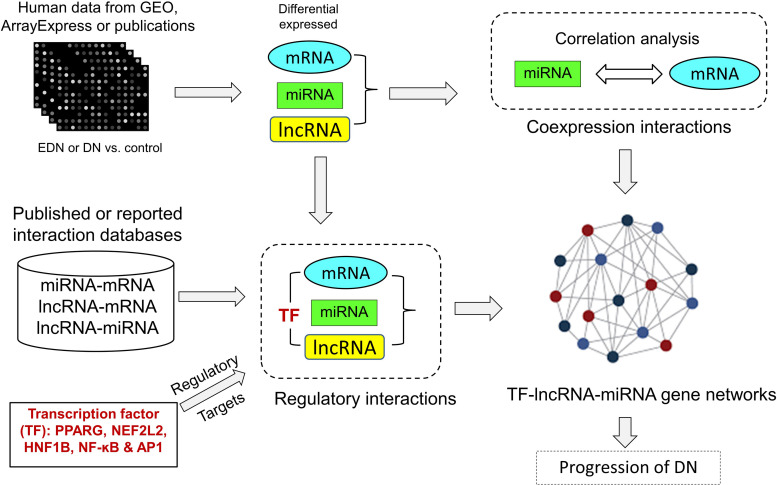
A pipeline analysis to identify interactions among TFs, lncRNAs, miRNAs, and mRNAs under DN. It includes three steps: (1) calculation of co-expression interactions between miRNAs and mRNAs from PCC analysis of DEG and DEM expression profiles obtained from EDN and DN datasets; (2) determination of interactive links among lncRNA–mRNAs or lncRNA–miRNAs generated by searching for lncRNA-targets (miRNA and mRNA) and miRNA–mRNAs; and (3) identification of TF–lncRNA–miRNA gene regulatory networks built from information generated in steps 1 and 2 and integrated with TF binding target data. PCC, Pearson correlation coefficient; DEG, differentially expressed genes/mRNAs; DEMs, differentially expressed miRNAs.

We developed an analysis pipeline capable of coordinating heterogeneous omics data to quantify and identify likely interactions among TFs, lncRNAs, miRNAs, and mRNAs and to organize TFs- and lncRNAs-mediated regulatory programs underlying pathogenesis of DN. Figure 1 shows a schematic and an overview of our pipeline procedures. For our analyses, we extracted genome-wide microarray and RNA-seq data of human mRNA and miRNA from published reports as described in *Methods*. The mRNA data included two sets for EDN and four sets for DN, while the miRNA data consisted of four sets for DN. From these datasets, we identified subsets of DEGs and DEMs implicated in both EDN and DN. To detect interactions between miRNAs and mRNAs, we calculated Pearson correction coefficient (PCC) using the expression data of DEGs and DEMs and searched miRNA target databases. We cataloged lncRNA-target resources and identified likely interactive relationships between lncRNAs and mRNAs or miRNAs. The three types of RNAs were selected and analyzed as being differentially expressed under EDN or DN. Finally, we incorporated TF binding target information and reconstructed TF–lncRNA–miRNA gene regulatory networks. The resulting multi-layered organizations are proposed to modulate cell signaling and biological functions that promote progression of DN.

### Expression Profiles of Differential mRNAs During EDN and DN

**FIGURE 2:**
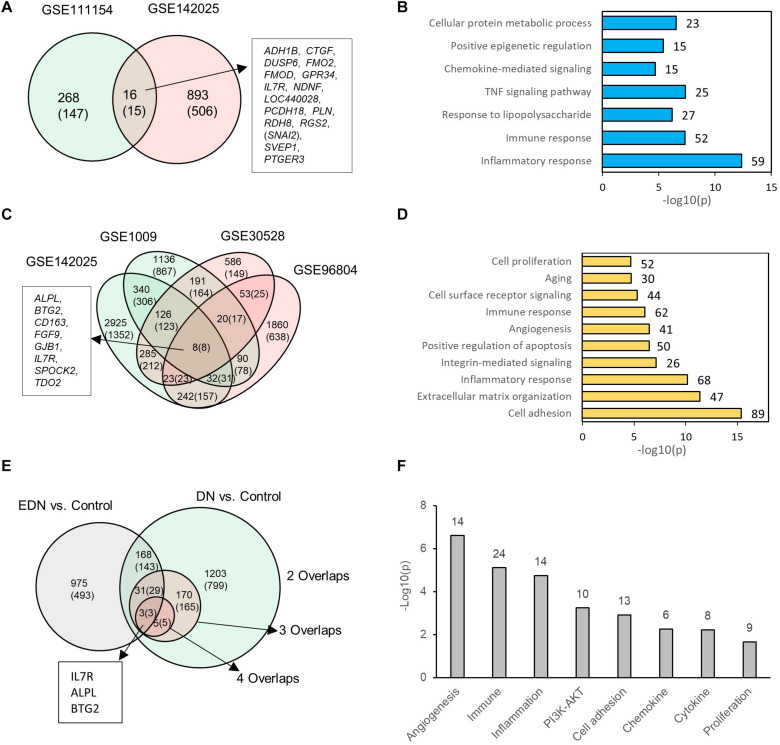
Differential expression profiles of mRNAs during EDN and DN. **(A)** a Venn diagram showing differentially expressed genes/mRNAs (DEGs) identified from two transcriptomic datasets of EDN. **(B)** Graph showing significant biological functions under EDN. **(C)** A Venn diagram showing DEGs identified from four transcriptomic datasets of DN. **(D)** Graph showing significant biological functions under DN. **(E)** A Venn diagram showing overlapping between EDN and DN. The “2, 3, and 4 overlaps” represents intersection of two, three, and four DN datasets, respectively. **(F)** Significantly enriched biological function of overlapped DEGs between EDN and DN. The number on each bar represents number of overlapped DEGs within this function category. In **A**, **C**, and **E**, numbers in the parentheses refer to number of DEGs with fold change at least 2.0.

We consolidated differentially expressed mRNAs or DEGs from gene expression datasets representative of EDN and DN populations (Supplementary Table S3). Two mRNA expression datasets related to EDN and control were analyzed. The result of Figure 2A shows 284 and 909 DEGs, respectively, generated from datasets GSE11154 and GSE142025 with fold changes of at least 1.5 by comparing EDN samples to control. There were 16 intersected DEGs, of which 15 displayed 2.0-fold changes, including *ADH1B*, *CTGF*, *DUSP6*, *FMO2*, *FMOD*, *GPR34*, *IL7R*, *NDNF*, *LOC440028*, *PCDH18*, *PLN*, *RDH8*, *RGS2*, *SVEP1*, and *PTGER3*. These DEGs, which may be informative and potential biomarkers of EDN, mainly involve inflammatory response, immune response, TNF pathway, and chemokine-mediated signaling, indicating early functional changes with DN (Figure 2B). An analysis of four microarray datasets of DN led to a different set of DEGs. These comparisons identified 1,410, 209, and 8 common genes corresponding to two, three, and four overlapped datasets, respectively. The eight DEGs (*ALPL*, *BTG2*, *CD163*, *FGF9*, *GJB1*, *IL7R*, *SPOCK2*, and *TDO2*) all had at least 2.0-fold changes in abundance (Figure 2C). Function annotation of the overlapped DEGs shows that cell adhesion, inflammatory response, immune response, angiogenesis, and cell surface receptor signaling were significantly enriched with DN status (Figure 2D).

To look for differences and commonalities that may serve as specific or common markers, respectively, of EDN and DN, we compared their DEGs. We obtained three sets of 168, 31, and 3 genes that could account for the common DEGs shared by at least one EDN with two, three, and four overlapped DN datasets, respectively (Figure 2E). Most of these overlapped DEGs had >2.0-fold changes in RNA abundance. We further annotated which biological processes or signaling pathways are significantly enriched with the overlapped DEGs. The resulting functions were angiogenesis, cell adhesion, inflammatory response, immune response, PI3K/AKT, cytokine, and chemokine (Figure 2F). This analysis points out a constitutive change of these biological functions during DN progression.

### Identification of miRNAs and mRNAs Interactions

**FIGURE 3:**
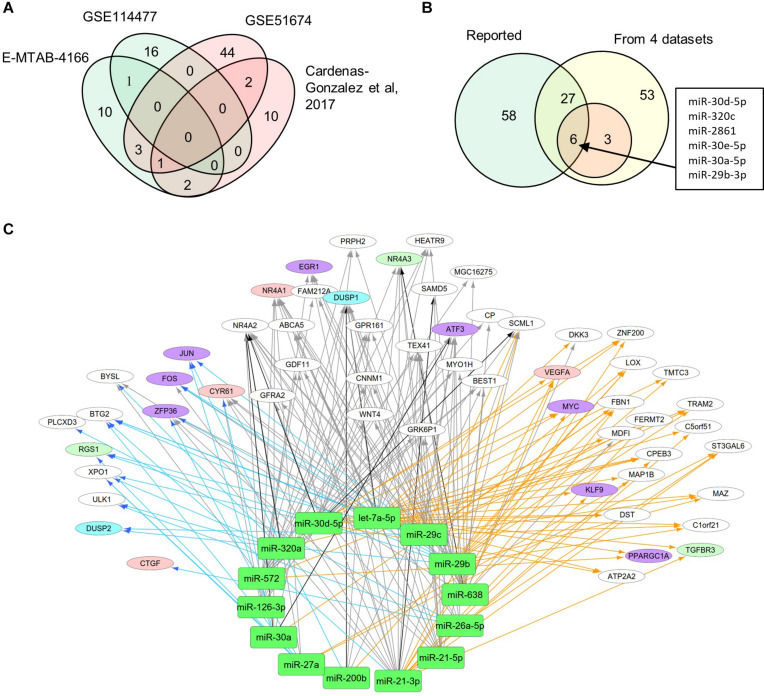
Differential expression profiles of miRNAs during DN. **(A)** Venn diagram showing differentially expressed miRNAs (DEMs) identified from two miRNA expression data of DN. There were two datasets from E-MTAB-4166 and Cardenas-Gonzalez et al. (2017). **(B)** Venn diagram showing overlap between the four miRNA expression datasets from Figure 2A and reported miRNAs from Supplementary Table S1. **(C)** a miRNA–mRNA interactive network. Blue, orange, and black/gray edges indicate miRNA–mRNA interactions identified under EDN, DN, and EDN + DN conditions, respectively, where black and gray edges also represent interactions generated based on miRNA target + PCC and only PCC, respectively. Circle nodes represent the DEGs involving biological processes apoptosis (light blue), angiogenesis (red), inflammation (orange), immune response (light green), and transcriptional regulation (purple).

MiRNAs likely have regulatory functions associated with the pathogenesis of DN. To test this postulate, we curated a list of 86 miRNAs that exhibits either altered expression or effects on expression of mRNAs during DN (Supplementary Table S1). We analyzed two sets of DEMs under DN and compared them with two published datasets (E-MTAB-4166; Cardenas-Gonzalez et al., 2017; Supplementary Table S4). A Venn diagram shows DEM overlap; however, none of the DEMs was shared by all four datasets. A total of nine miRNAs were differentially expressed in at least two datasets, including miR-30d-5p, which was identified from three datasets (Figure 3A). As compared to the reported miRNAs (Supplementary Table S1), we detected six of the nine miRNAs that are consistent, including miR-30d-5p, miR-320c, miR-2861, miR-30e-5p, miR-30a-5p, and miR-29b-3p (Figure 3B). These miRNAs may be representative of frequently altered miRNAs during DN.

A typical miRNA–mRNA interaction involves post-transcriptional control of transcript stability or processing by miRNAs. We selected a set of 18 miRNAs (from Figure 3B and Supplementary Table S1) implicated in the pathogenesis of DN and which may be involved in post-transcriptional control. To seek their interactive relationships with mRNAs under EDN and DN, we analyzed the co-expression network of miRNA and mRNA, which has become an effective method to identify regulatory function of miRNAs. We then used a revised Pearson correlation method able to compare expression data between miRNAs and mRNAs from different numbers of samples. We identified a total of 461 and 889 DEGs from EDN and DN, respectively, that had a negative correlation with the 18 miRNAs. In this study, we considered a negative correlation to be indicative of RNA degradation and post-transcriptional control. Table 1 shows the number of inversely correlated DEGs for every miRNA. Noticeably, miR-21, -30d-5p, -572, and -638 were co-expressed with over 80 DEGs in either EDN or DN. Of note, these four miRNAs, as well as let-7a-5p and miR-126-3p, were co-expressed with at least 10 DEGs under both conditions. Next, we combined two main resources of miRNA-targets, miRTarBase and miRecords, to identify mRNAs that were likely miRNA targets. Targets were chosen only if they were experimentally validated and/or those genes were predicted from at least 4 of 11 available miRNA target prediction methods. A total of 284, 164, and 60 DEGs under EDN were found as targets of at least 1, 2, and 4 of the 18 miRNAs, respectively. By contrast, under DN, these numbers were 2,477, 1,532, and 575, respectively, indicating a greater number of target mRNAs for DN than for EDN.

**TABLE 1:** Interactions between mRNAs and miRNAs during EDN and DN.

**miRNA**	**EDN**				**DN**				**EDN + DN**		
	**Target**	**PCC**	**Target + PCC**	**Function**	**Target**	**PCC**	**Target + PCC**	**Function**	**PCC**	**Target + PCC**	**Function**
let-7a-5p	18	30	10	AG, CA, IM, PL	52	41	23	IM, PI3K	18	2	AG, IM
miR-10a-5p	1	2	1								
miR-126-3p	1	13	1	AP, IF	2	11	0	AG, IM, CA	11		AG, IM, CA
miR-192	45	13	1	IF, PL							
miR-200a	31	12	1	AG, PI3K							
miR-200b	38	9	0		376	12	1	AG, IF	3	0	
miR-21-3p	30	123	6	CC, PL	303	534	40	AG, AP,	39	3	CC
miR-21-5p	30	117	8	CC, PL	303	357	33	AG	24	1	AG, IM, CA
miR-26a-5p	48	38	0	AG, IM, IF, PL	386	30	1	AP, CA	5	0	AG
miR-27a	53	22	4	IM, PL	435	10	3	AG, CA,	2	0	AG
miR-29b-3p	39	49	6	IM	327	72	7	AG, IM, CA	9	0	AG, CA
miR-29c-3p	36	53	9	IM, PL	323	44	1	AD, IM, CA	9	1	AG
miR-30a-5p	55	32	7	AG	623	10	2	AG, PL	4	2	AG, CA
miR-30c-5p	46	25	1	AG	480						
miR-30d-5p	42	53	0	AG, PL,	450	131	8	PL	12	0	AG, PL
miR-320a	22	42	1	AG, IM	241	0	0	AG, IM	22	0	AG, AP
miR-320c	4	10	0	AP	38	0	0	AG, AP	4	0	AP
miR-572	7	64	0	AG	63	356	2	AP, IF, IM, CA	19	0	CA, IF
miR-638	7	73	0	AG, CA	112	218	0	IM, CA, IF	18	0	CA

**Table shows the number of identified miRNA–mRNA interactions based on target search and PCC. Targets were derived from mirTarbase and miRecords. PCC (Pearson correlation coefficient) refers to correlation between expression of miRNAs and mRNAs with p <0.01. For targeted function: CC, cell cycle; CA, cell adhesion; AG, angiogenesis; AP, apoptosis; PL, proliferation; IF, inflammation; IM, immune response.**

In order to obtain high-confidence miRNA–mRNA interactions, we integrated the co-expression and target mRNA approaches. This led to the identification of probable miRNA–mRNA interactions that are likely indicative of potential regulatory relationships (Table 1). In particular, let-7a-5p, miR-21-5p, -30d-5p, and -29c-3p miRNAs were found to have more mRNA targets under EDN than other miRNAs. Similarly, these four miRNAs interact with more DEGs under DN, suggesting they play a central role in regulating DEGs during the entirety of DN pathogenesis. Our interactive regulatory network consisting of miRNAs and their targeted mRNAs are shown in Figure 3C. Among these are miRNAs (let-7a, miR-21, -30a, -30d, -26a, -29b, -29c, -200b, -320a, -572, and -638) which are predicted to interact differentially with targeted mRNAs in EDN, DN, and EDN + DN. The gene transcripts targeted by these miRNAs were mainly associated with transcriptional regulation, angiogenesis, apoptosis, immune response, and cell proliferation.

### Identification of lncRNAs and miRNA/mRNA Interactions

**FIGURE 4:**
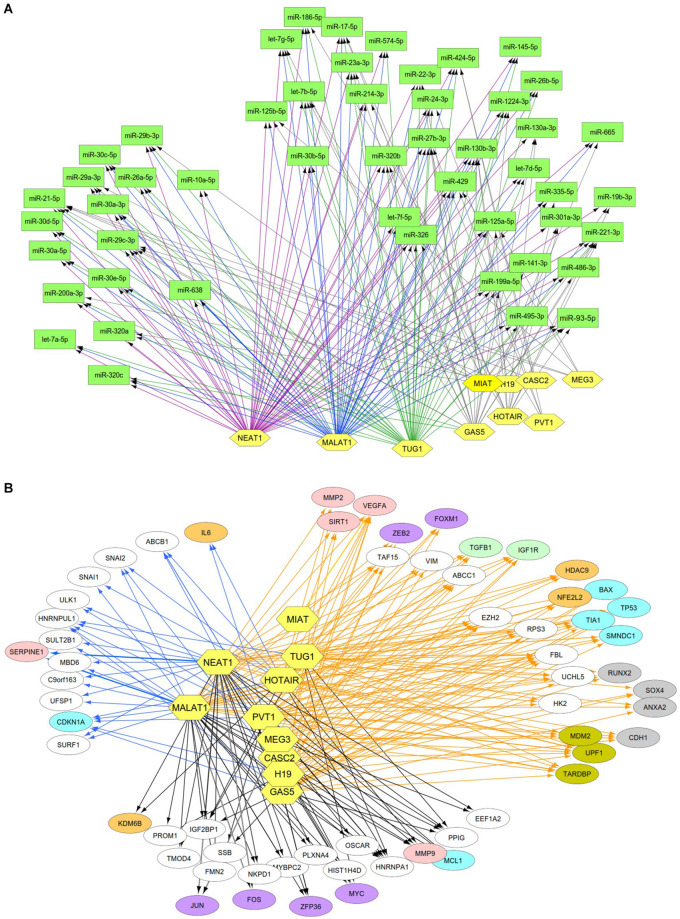
Interactive networks of lncRNA-targets (miRNAs or mRNAs) during DN. **(A)** Interactive networks of lncRNA–miRNAs. **(B)** Interactive networks of lncRNA–mRNAs under EDN and DN. Blue, orange, and black edges indicate lncRNA–mRNA interactions identified under EDN, DN, and EDN + DN conditions, respectively. Yellow nodes stand for lncRNAs. Circle nodes represent the DEGs involving biological processes apoptosis (light blue), angiogenesis (red), inflammation (orange), immune response (light green), cell cycle (brown), and transcriptional regulation (purple).

Previous studies reported that DN was associated with the over-expression of MALAT1, PVT1, GAS5, H19, MEG3, NEAT1, and HOTAIR and the under-expression of MIAT, TUG1, and CASC2. Since these lncRNAs may play an important regulatory role in DN (Supplementary Table S2), we postulated that these lncRNAs affect DN-related biological functions by targeting miRNAs or mRNAs. To identify possible interactive connections between lncRNAs and targets (miRNAs and mRNAs), we searched four databases (LncBases, NPInter, LncTarD, and starBase). These databases facilitate the identification of lncRNA–miRNA and lncRNA–mRNA interactions that are supported by experimentally validated and high-throughput experimental tests. Because some lncRNAs in the four databases have few or no known human targets that match our DEGs or DEMs, we selected 10 lncRNAs from Supplementary Table S2 that were used to further our interactive analysis (Table 2). We found that a total of 91 DEMs or reported miRNAs interact with the 10 selected lncRNAs used in these analyses (Supplementary Table S4). MALAT1, TUG1, GAS5, and NEAT1 were found to have 66, 47, 23, and 83 miRNA targets, respectively (Table 2). In particular, MALAT1, TUG1, and NEAT1 co-target 39 miRNAs. Among the targeted miRNAs, miR-21-5p and -29c-3p can be regulated by at least six lncRNAs. Other miRNAs listed in Table 1 may also be targets of three or four lncRNAs, including miR-200a-3p, -29b-3p, -320c, let-7a-5p, -130a-3p, -26a-5p, -30a-5p, -30c-5p, -30d-5p, and -320a (Supplementary Table S4 and Figure 4A). Integrated with these interactive links, we constructed a lncRNA–miRNA regulatory network of DN (Figure 4A).

**TABLE 2:** Interactions between lncRNA and mRNAs or miRNAs.

**lncRNA**	**miRNA**		**mRNA**			
	**All**	**Main targets**	**EDN**	**DN**	**EDN + DN**	**Targeted function**
CASC2	4		1	6	1	CC, CA
GAS5	23	miR-320c	7	34	5	AP, AG, CC, PL
H19	3		11	45	6	IF, AP, AG, IM, PL, CC, CA
HOTAIR	14		15	53	10	IM
MALAT1	66	miR-30d-5p, -10a-5p, -29b-3p, -30a-5p, -30e-5p, -320c -638, -572	47	386	29	AP, IM, PL, PI3K-AKT
MEG3	6		5	36	3	AG
MIAT	10	miR-29b-3p	1	7	1	AG, IF
NEAT1	83	miR-30d-5p, -320c, -29b-3p, -30a-5p, -30e-5p, -10a-5p, -638	93	755	63	CC, CA, IM, PI3K-AKT
PVT1	7		12	33	8	PI3K-AKT
TUG1	47	miR-30d-5p, -29b-3p, -30a-5p, -30e-5p, -320c	7	59	6	CC, AP, IM, PL, PI3K-AKT

**Table shows the number of identified lncRNA–miRNA and lncRNA–mRNA interactions based on lncRNA related target databases. For targeted function: CC, cell cycle; CA, cell adhesion; AG, angiogenesis; AP, apoptosis; PL, proliferation; IF, inflammation; IM, immune response.**

Having identified likely interactions between lncRNAs and miRNAs, we constructed interactive relationships between lncRNAs and mRNAs. In total, we detected 137 DEGs targeted by at least 1 of the 10 lncRNAs under EDN (Supplementary Table S3). Six genes *HNRNPA1*, *IGF2BP1*, *CDKN1A*, *MYC*, *HNRNPUL1*, and *PPIG* can be targeted by at least five lncRNAs. It is notable that MALAT1 and NEAT1 can interact with 47 and 93 DEGs, respectively, a number much greater than that observed with the other eight lncRNAs analyzed (Table 2). Altogether, a total of 1,018 DEGs were targeted by at least one lncRNA, showing a larger number of lncRNA–mRNA regulatory relations under DN. Similarly, when compared to the other eight lncRNAs, MALAT1 and NEAT1 regulated a much higher number of DEGs (386 and 755 under DN, and 29 and 63 under both EDN and DN, respectively) (Table 2). They can commonly target 243 DEGs (Supplementary Table S3), a finding that suggests that the two lncRNAs may be critical modulators in the pathogenesis of DN. By combining EDN with DN, we constructed a complete lncRNA–mRNA regulatory network (Figure 4B). It is noteworthy that the main biological functions of the network under EDN and DN are transcriptional regulation, angiogenesis, apoptosis, inflammatory response, and immune response, among others.

### Identification of TFs–lncRNAs–miRNA–mRNA Interactions

NF-κB (NFKB1 and RELA), NEF2L2, HNF1B, PPARG, and AP1 are believed to be important TFs in the pathogenesis of DN. They regulate expression of target genes by interacting with cis-regulatory regions around these genes. To predict putative target genes of these TFs, we identified consensus TF binding sites that were conserved in both human and mouse gene promoters. Under EDN and DN, there were 157 and 977 DEGs, respectively, that contained cis-elements that may be regulated by at least two TFs (Supplementary Table S3). Under EDN, *EGR3* and *NR4A2* may be regulated by at least five TFs. Under DN, *BDNF* and *PTCHD1* are targets of all of the TFs analyzed.

**FIGURE 5:**
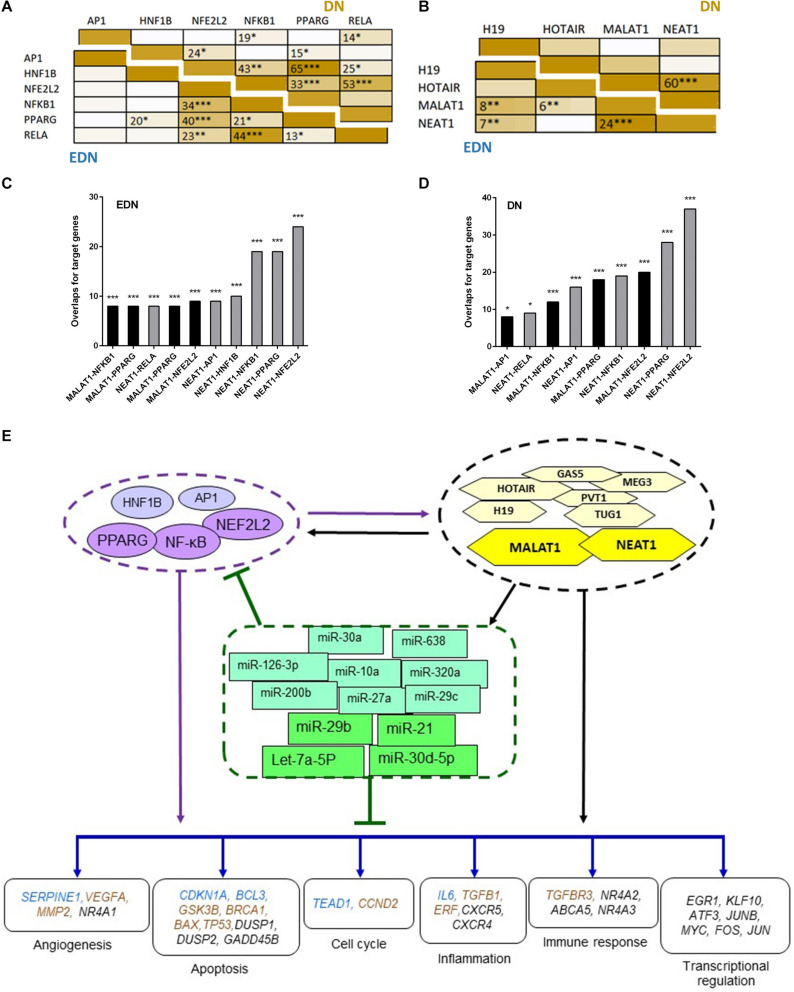
Interactive networks of TF–lncRNA–miRNA–mRNA under DN. **(A)** Pairwise comparison of TF targets based on enrichment *p*-value of TF target overlapping. The darker the color, the lower the *p*-value. **(B)** Pairwise comparison of lncRNA targets based on enrichment *p*-value of lncRNA target overlapping. **(C,D)** Comparison of the target DEGs of TFs and lncRNAs by calculating enrichment *p*-value under EDN **(C)** and DN **(D)**. *Y* axis refers to overlapped number of target genes. **(E)** a model showing multi-level regulatory relationships among TFs, lncRNAs, miRNAs, and downstream target genes. Yellow and purple nodes stand for lncRNAs and TFs, respectively. Genes in blue, orange, and black fonts indicate DEGs under EDN, DN, and EDN + DN, respectively. *, **, and *** indicate enrichment *p* < 10^–2^, 10^–4^, and 10^–6^, respectively.

To define whether two TFs can co-regulate target genes, we performed hypergeometric analysis using overlapped target genes of the two TFs, where *p*-values are used to evaluate significance of enrichment among the TF targets. We compared target DEGs of six TFs under EDN or DN. As shown in Figure 5A, there were significant overlaps of the targeted DEGs under DN between NFE2L2 and PPARG, HNF1B, or NF-κB. Other interactions, like NFKB1–PPARG, AP1–NF-κB, and HNF1B–PPARG also displayed significant co-regulatory relationships. Similar to DN, we found significant interactions of nearly identical TF pairs under EDN. We then compared interactions between lncRNAs and found a highly significant interaction between MALAT1 and NEAT1 under both EDN and DN (Figure 5B). Finally, we determined the significance of these interactions between two lncRNAs (MALAT1 and NEAT1) and four TFs (NFE2L2, AP1, PPARG, and NF-κB) under the two DN conditions. The results are shown in Figures 5C,D.

It is known that lncRNAs sponge and regulate miRNA expression or compete with mRNA for miRNA, while crosstalk among diverse RNA species involving mRNAs, lncRNAs, and miRNAs is important for gene regulatory complexity (Tay et al., 2014). MiRNAs can bind to numerous target RNA transcripts, which has led to the hypothesis that target transcripts may compete for binding with shared miRNAs and thus act as competing endogenous RNAs (ceRNAs) (Salmena et al., 2011; Tay et al., 2014). Here, we identified DEGs that have common pools of miRNAs which target these mRNAs and negatively correlate with the expression of DEG, and may be targets of lncRNAs (Table 3). The result shows that five DEGs (*BTG2*, *CPEB3*, *TGFBR3*, *KLF9*, and *C5orf51*) are targeted by let-7a-5p and miR-21. These miRNAs in turn are targets of four lncRNAs (MALAT1, NEAT, TUG1, and MIAT). Similarly, miR-30a and miR-29c are shared by *JUN* and lncRNAs (MALAT1, NEAT, and TUG1). This result suggests that the predicted ceRNAs represent a regulatory layer linking the three types of RNAs in DN.

**TABLE 3:** List of ceRNAs interactions with miRNAs and lncRNAs.

**ceRNAs (mRNAs)**	**Shared miRNAs**	**LncRNA to targeting miRNAs**
*C1orf21*, *SCML1*, *LOX*, *TRAM2*, *SLC16A7*, *FERMT2*, *ZFP36L1*, *DIAPH2*, *FOS*, *DUSP2*	miR-29b, miR-29c	MALAT1, MIAT, NEAT, TUG1
*NR4A2*, *ATF3*, *CYR61*	miR-21, miR-30a	MALAT1, NEAT, TUG1
*BTG2*, *ZFP36L1*, *C1orf21*, *TMTC3*	let-7a-5p, miR-29b, miR-29c	MALAT1, NEAT, TUG1
*BTG2*, *CPEB3*, *TGFBR3*, *KLF9*, *C5orf51*	let-7a-5p, miR-21	MALAT1, NEAT, TUG1, MIAT
*JUN*	miR-30a, miR-29c	MALAT1, NEAT, TUG1
*BTG2*, *RGS1*, *ZFP36*	miR-27a, miR-29b	MALAT1, MIAT, NEAT, TUG1
*RGS1*	miR-27a, miR-29b, miR-29c	MALAT1, NEAT, TUG1

**Table shows the identified ceRNAs targeted by the pool of shared miRNAs that reversely corrected with the ceRNAs.**

To construct regulatory relationships among TFs, lncRNAs, and miRNAs during the progression of DN, we assembled all three types of interactive connections, miRNA–mRNA (Figure 3C), lncRNA–miRNA (Figure 4A), and lncRNA–mRNA (Figure 4B). By combining these connections with TF target genes, we established a model of TF–lncRNA–miRNA–mRNA regulatory networks (Figure 5E). LncRNAs MALAT1 and NEAT1 are likely to cooperate with TFs (NF-κB, PPARG, and NFE2L2) and with miRNAs (miR-30d-5p, miR-21, miR-29b, let-7a-5p, etc.) to regulate expression of genes associated with a variety of biological processes, including angiogenesis, apoptosis, inflammation, immune response, and transcriptional regulation in EDN. Other TFs, lncRNAs, and miRNAs form an expanded gene regulatory network that involves more function or cell signaling responsible for progressive DN pathogenesis.

## Discussion

Diabetes-induced kidney disease is a serious global public health problem, but the molecular mechanism responsible for the pathogenesis of DN from early to late stages remains unclear. The regulatory importance of previously identified TFs, miRNAs, and lncRNAs in the development or progression of DN has been reported (Bernardi et al., 2019; Guo et al., 2019; Sankrityayan et al., 2019), but when several researchers have tried to identify interactions among these factors and the related mechanisms of DN pathogenesis (Kato, 2018), the results have proven inconclusive. Here we provide evidence showing that the combinational performance of TFs, miRNAs, and lncRNAs factors is central to the regulatory mechanisms that control gene expression associated with DN. Specifically, we described the development of a network structure pipeline that permitted us to perform an integrated analysis of omics data with factor-target resources. We constructed a TF–miRNA–lncRNA gene regulatory network to uncover putative key regulatory factors and target genes related with EDN and DN. Our analyses indicated that the two lncRNAs (MALAT1 and NEAT1) and the three TFs (NF-κB, NFE2L2, and PPARG) may play cooperative regulatory roles with a set of miRNAs to control downstream DEGs. These results represent the first report describing how TF and ncRNA interactions contribute to the regulatory control of DN in an integrated manner.

LncRNAs represent a type of ncRNA comprising transcripts longer than 200 nucleotides. They have been found to regulate patterns of protein expression through diverse biological interactions among lncRNAs and proteins, lncRNAs and mRNAs, and lncRNAs and ncRNAs (Li et al., 2014). As a result, the construction of maps of putative biological interaction networks will likely contribute to our understanding of biological functions and mechanisms controlled by lncRNAs. Previous reports described a number of lncRNAs (e.g., H19, MALAT1, NEAT1, TUG1, and MEG3) implicated in the development and progression of DN either via direct mediators of pathogenesis or as indirect mediators of nephropathic signaling pathways involving TNFα, TGFβ1, NF-κB, AP1, and GSK3β (Guo et al., 2019). The mechanisms whereby lncRNAs interact with these and other regulators to affect DN progression, however, remain unclear. In this study, we identified interactions between lncRNAs and both mRNAs and miRNAs. In EDN, some lncRNA-targeted DEGs are related specifically to inflammation (*IL6*, *CXCR4*, and *CXCR5*), apoptosis (*CDKN1A*), angiogenesis (*SERPINE1*), and transcriptional control (*FOS*, *JUN*, *JUNB*, *MYC*, *ATF3*, and *EGR1*). These results suggest that the up-regulation of MALAT1 and NEAT1 among other lncRNAs mediate DN-related biological processes during EDN. Notably, several previously identified DN-associated TFs (NF-κB, NEF2L2, and PPARG) can bind to promoters of these DEGs, including the TF genes *JUN*, *FOS*, *JUNB*, and *MYC*. We also found that the target DEGs (*VEGFR*, *GSK3B*, *TP53*, *BAX*, *TGFR1*, *TGFBR3*, and *BRCA1*) identified only in DN are associated with the biological processes similar to those observed in EDN. Previous studies have validated lncRNA–TF interactions, for example, MALAT1 and NF-κB (Ding et al., 2018), TUG1 and PPARG in mesangial cells of DN (Duan et al., 2017), upregulated PVT1 and the JNK/NF-κB signaling pathways. In diabetic foot, MALAT and NEF2L2 forms a loop regulation of angiogenesis with MALAT1/HIF1A (Jayasuriya et al., 2020). NEAT1 suppresses miR-124 and NF-κB pathway. Similarly, several lncRNA–miRNA interactions were confirmed by experimental testing, including MALAT1-miR-21 (Huang et al., 2020), MALAT1-miR-30 (Yi et al., 2019), NEAT1-miR-21 (Quan et al., 2020), and NEAT1-let-7a (Liu et al., 2018). These results provide experimental support for our analysis on the cooperative function of lncRNAs in DN development and progression.

The role of miRNAs in DN may be multi-factorial. First, it is clear that miRNAs are differentially expressed between healthy control and early and advanced stages of DN. This has led to the postulate that let-7a, miR-21, and miR-29b play important roles in the regulation of DN development and progression (Kato, 2018). Second, actions of ceRNAs represent a novel mechanism of gene regulation that mediates aberrant expression of mRNAs and miRNAs. Several ceRNAs have been shown to interact with TFs, miRNAs, or lncRNAs that result in altered gene expression during disease and cancer progression (Tay et al., 2014). In DN, several studies reported function of lncRNA as ceRNAs in affecting signaling pathways (Chen et al., 2019; Ji et al., 2019). In this study, we identified DEGs as ceRNAs that have negative coexpression with the shared miRNAs, let-7a-5p, miR-21, miR-30a, miR-29b, etc. These miRNA pools may be targets of lncRNAs. It is also noteworthy that these DEGs can be regulated by TFs NF-κB, PPARG, and NEF2L2. Therefore, our analysis of ceRNAs also provides evidence to support the interactions among the DEGs and lncRNAs described in this study. ceRNAs are likely integral to the control of crosstalk among the shared miRNAs.

As compared to previous studies, the most prominent innovation of our study is to establish a multi-level hierarchical gene regulatory network formed by interactions among TFs, lncRNAs, and miRNAs associated with DN pathogenesis and progression. The integrated pipeline established here confirms that different regulatory programs of DEGs and DEMs are involved in diverse biological processes and pathways associated with DN. Among the array of possible regulatory factors, we identified two lncRNAs (MALAT1 and NEAT1) and three TFs (NF-κB, NEF2L2, and PPARG) that cooperatively work together with miRNAs to become controllers of DN onset and progression. LncRNAs MALAT1 and NEAT1 are thus likely to serve as biomarkers for early diagnosis or prognosis of DN or as therapeutic targets for suppressing progression of established DN. These five factors are also putative targets of DN treatment; however, detailed functional analysis of these factors in DN requires further study in the future. Finally, having established a pipeline for describing interactions between TFs and ncRNAs, the use of high-throughput datasets to describe regulatory interactions should be applicable to any system where data are available. Integrated analysis of multi-omics data thus will provide a basis for inferring the interplays among TFs, lncRNAs, and miRNAs and for elucidating the regulatory mechanisms underlying DN as well as other diseases.

## Data Availability Statement

All datasets generated for this study are included in the article/Supplementary Material.

## Author Contributions

LC, BW, SW, YX, BZ, XC, and TZ performed data processing and analyzed data. LC, JC, BY, and BW participated in writing the manuscript. BY, RL, and T-WL contributed to discussion and critical evaluation of the manuscript. JC and BY designed the study. All authors read and approved the manuscript.

## Conflict of Interest

The authors declare that the research was conducted in the absence of any commercial or financial relationships that could be construed as a potential conflict of interest.

## Footnotes

1 http://bigdata.ibp.ac.cn/npinter

2 http://biocc.hrbmu.edu.cn/LncTarD

3 http://starbase.sysu.edu.cn/
