# Supplementary material for: Identification of Cooperative Gene Regulation Among Transcription Factors, LncRNAs, and MicroRNAs in Diabetic Nephropathy Progression
Source: Front Genet. 2020 Sep 1;11:1008. doi: 10.3389/fgene.2020.01008 (PMC7490338; doi:10.3389/fgene.2020.01008)
Supplement: Supplementary file 1 [file Table_1.DOCX]

**Table S1. Altered or dysregulated miRNAs reported in human DN studies**

| miRNA | Upregulated | Downregulated | References |
| --- | --- | --- | --- |
| let-7a-5p |  | 3 | (1-3) |
| miR-10 |  | 1 | (1) |
| miR-100-5p | 1 |  | (4) |
| miR-1224-3p | 1 |  | (5) |
| miR-122-5p | 2 |  | (1, 6) |
| miR-1247-5p |  |  | (1) |
| miR-126 |  | 1 | (7) |
| miR-126-3p | 3 |  | (1, 2, 8) |
| miR-1303 | 1 |  | (9) |
| miR-130a | 1 |  | (10) |
| miR-130b | 2 | 3 | (11-15) |
| miR-130b-3p |  |  | (11) |
| miR-135a-5p | 2 |  | (4, 8) |
| miR-141-3p | 1 |  | (5) |
| miR-145 | 1 |  | (10) |
| miR-146a | 2 | 3 | (16-20) |
| miR-155 | 2 | 4 | (10, 21-25) |
| miR-17-5p | 1 |  | (1) |
| miR-181a | 3 |  | (26-28) |
| miR-186-5p | 2 |  | (4, 29) |
| miR-1912 | 1 |  | (5) |
| miR-1915-3p | 1 | 1 | (4, 30) |
| miR-192 |  | 3 | (31-33) |
| miR-199a-5p | 1 |  | (1) |
| miR-200b-3p |  | 1 | (1) |
| miR-200a-3p |  | 1 | (4) |
| miR-200c-3p |  | 1 | (8) |
| miR-21-5p | 4 |  | (2, 8, 34, 35) |
| miR-214-3p | 2 |  | (16, 36) |
| miR-215 | 2 |  | (31, 37) |
| miR-21 | 3 |  | (11, 35, 38) |
| miR-216a |  | 2 | (39, 40) |
| miR-221-3p |  | 1 | (5) |
| miR-23 |  |  | (1) |
| miR-23b |  | 3 | (41-43) |
| miR-25-3p |  | 2 | (4, 44) |
| miR-26a-5p |  | 3 | (2, 3, 45) |
| miR-27a | 3 |  | (46-48) |
| miR-2861 |  | 2 | (4, 49) |
| miR-29a-3p |  | 4 | (50-53) |
| miR-29b-1-5p | 1 |  | (5) |
| miR-29c | 3 |  | (54-56) |
| miR-29c-3p |  | 3 | (2, 8, 57) |
| miR-30 |  |  | (1, 22) |
| miR-302a-3p | 2 |  | (1, 58) |
| miR-30a |  | 1 | (59) |
| miR-30a-5p | 1 |  | (1) |
| miR-30c-5p | 4 |  | (2, 3, 8, 30) |
| miR-30d-5p |  | 2 | (4, 49) |
| miR-30e-5p |  | 1 | (49) |
| miR-320a | 2 |  | (4, 60) |
| miR-320c | 2 |  | (3, 49) |
| miR-323b-5p |  | 1 | (5) |
| miR-326 | 2 |  | (22, 26) |
| miR-335-5p | 1 |  | (5) |
| miR-377 | 2 |  | (39, 61) |
| miR-424 |  | 1 | (10) |
| miR-424-5p | 1 |  | (5) |
| miR-4270 | 1 |  | (49) |
| miR-429 | 1 |  | (5) |
| miR-4532 |  | 1 | (4) |
| miR-4536-3p | 1 |  | (4) |
| miR-486-3p | 1 |  | (5) |
| miR-495 | 1 |  | (1) |
| miR-495-3p |  | 1 | (4) |
| miR-501-5p | 1 |  | (4) |
| miR-548c-3p | 1 |  | (1) |
| miR-548o-3p | 1 |  | (1) |
| miR-552 | 1 |  | (5) |
| miR-571 | 1 |  | (9) |
| miR-574-3p |  | 2 | (22, 26) |
| miR-6068 | 1 |  | (4) |
| miR-616-5p | 1 |  | (1) |
| miR-619 | 1 |  | (5) |
| miR-640 | 1 |  | (1) |
| miR-645 | 1 |  | (1) |
| miR-661 | 1 |  | (9) |
| miR-665 | 1 |  | (1) |
| miR-6747 | 1 |  | (4) |
| miR-767-3p | 1 |  | (1) |
| miR-770-5p | 2 |  | (1, 62) |
| miR-7a-5p | 1 |  | (1) |
| miR-7b-3p | 1 |  | (1) |
| miR-886 |  |  | (31) |
| miR-892b | 1 |  | (9) |
| miR-93-5p | 1 |  | (4) |

Reference

1. Argyropoulos C, Wang K, Bernardo J, Ellis D, Orchard T, Galas D, et al. Urinary MicroRNA Profiling Predicts the Development of Microalbuminuria in Patients with Type 1 Diabetes. *J Clin Med.* 2015;4(7):1498-517.

2. Krupa A, Jenkins R, Luo DD, Lewis A, Phillips A, and Fraser D. Loss of MicroRNA-192 promotes fibrogenesis in diabetic nephropathy. *J Am Soc Nephrol.* 2010;21(3):438-47.

3. Zhou J, Peng R, Li T, Luo X, Peng H, Zha H, et al. A potentially functional polymorphism in the regulatory region of let-7a-2 is associated with an increased risk for diabetic nephropathy. *Gene.* 2013;527(2):456-61.

4. Gholaminejad A, Abdul Tehrani H, and Gholami Fesharaki M. Identification of candidate microRNA biomarkers in diabetic nephropathy: a meta-analysis of profiling studies. *J Nephrol.* 2018;31(6):813-31.

5. Argyropoulos C, Wang K, McClarty S, Huang D, Bernardo J, Ellis D, et al. Urinary microRNA profiling in the nephropathy of type 1 diabetes. *PLoS One.* 2013;8(1):e54662.

6. Regmi A, Liu G, Zhong X, Hu S, Ma R, Gou L, et al. Evaluation of Serum microRNAs in Patients with Diabetic Kidney Disease: A Nested Case-Controlled Study and Bioinformatics Analysis. *Med Sci Monit.* 2019;25:1699-708.

7. Al-Kafaji G, Al-Mahroos G, Al-Muhtaresh HA, Skrypnyk C, Sabry MA, and Ramadan AR. Decreased expression of circulating microRNA-126 in patients with type 2 diabetic nephropathy: A potential blood-based biomarker. *Exp Ther Med.* 2016;12(2):815-22.

8. He F, Peng F, Xia X, Zhao C, Luo Q, Guan W, et al. MiR-135a promotes renal fibrosis in diabetic nephropathy by regulating TRPC1. *Diabetologia.* 2014;57(8):1726-36.

9. Wang C, Wan S, Yang T, Niu D, Zhang A, Yang C, et al. Increased serum microRNAs are closely associated with the presence of microvascular complications in type 2 diabetes mellitus. *Sci Rep.* 2016;6:20032.

10. Barutta F, Tricarico M Fau - Corbelli A, Corbelli A Fau - Annaratone L, Annaratone L Fau - Pinach S, Pinach S Fau - Grimaldi S, Grimaldi S Fau - Bruno G, et al. Urinary exosomal microRNAs in incipient diabetic nephropathy. *PLoS One.* 2013;8(11):e73798.

11. Sankrityayan H, Kulkarni YA, and Gaikwad AB. Diabetic nephropathy: The regulatory interplay between epigenetics and microRNAs. *Pharmacol Res.* 2019;141:574-85.

12. Ma Y, Shi J, Wang F, Li S, Wang J, Zhu C, et al. MiR-130b increases fibrosis of HMC cells by regulating the TGF-beta1 pathway in diabetic nephropathy. *J Cell Biochem.* 2019;120(3):4044-56.

13. Motawi TK, Shehata NI, ElNokeety MM, and El-Emady YF. Potential serum biomarkers for early detection of diabetic nephropathy. *Diabetes Res Clin Pract.* 2018;136:150-8.

14. Bai X, Geng J, Zhou Z, Tian J, and Li X. MicroRNA-130b improves renal tubulointerstitial fibrosis via repression of Snail-induced epithelial-mesenchymal transition in diabetic nephropathy. *Sci Rep.* 2016;6:20475.

15. Lv C, Zhou YH, Wu C, Shao Y, Lu CL, and Wang QY. The changes in miR-130b levels in human serum and the correlation with the severity of diabetic nephropathy. *Diabetes Metab Res Rev.* 2015;31(7):717-24.

16. Bhatt K, Lanting LL, Jia Y, Yadav S, Reddy MA, Magilnick N, et al. Anti-Inflammatory Role of MicroRNA-146a in the Pathogenesis of Diabetic Nephropathy. *J Am Soc Nephrol.* 2016;27(8):2277-88.

17. Wan RJ, and Li YH. MicroRNA146a/NAPDH oxidase4 decreases reactive oxygen species generation and inflammation in a diabetic nephropathy model. *Mol Med Rep.* 2018;17(3):4759-66.

18. Lee HW, Khan SQ, Khaliqdina S, Altintas MM, Grahammer F, Zhao JL, et al. Absence of miR-146a in Podocytes Increases Risk of Diabetic Glomerulopathy via Up-regulation of ErbB4 and Notch-1. *J Biol Chem.* 2017;292(2):732-47.

19. Huang Y, Liu Y, Li L, Su B, Yang L, Fan W, et al. Involvement of inflammation-related miR-155 and miR-146a in diabetic nephropathy: implications for glomerular endothelial injury. *BMC Nephrol.* 2014;15:142.

20. Alipour MR, Khamaneh Am Fau - Yousefzadeh N, Yousefzadeh N Fau - Mohammad-nejad D, Mohammad-nejad D Fau - Soufi FG, and Soufi FG. Upregulation of microRNA-146a was not accompanied by downregulation of pro-inflammatory markers in diabetic kidney. *Mol Biol Rep.* 2013;40(11):6477-83.

21. Akhbari M, Khalili M, Shahrabi-Farahani M, Biglari A, and Bandarian F. Expression Level of Circulating Cell Free miR-155 Gene in Serum of Patients with Diabetic Nephropathy. *Clin Lab.* 2019;65(8).

22. Wang LP, Gao YZ, Song B, Yu G, Chen H, Zhang ZW, et al. MicroRNAs in the Progress of Diabetic Nephropathy: A Systematic Review and Meta-Analysis. *Evid Based Complement Alternat Med.* 2019;2019:3513179.

23. Beltrami C, Simpson K, Jesky M, Wonnacott A, Carrington C, Holmans P, et al. Association of Elevated Urinary miR-126, miR-155, and miR-29b with Diabetic Kidney Disease. *Am J Pathol.* 2018;188(9):1982-92.

24. Wang Y, Zheng ZJ, Jia YJ, Yang YL, and Xue YM. Role of p53/miR-155-5p/sirt1 loop in renal tubular injury of diabetic kidney disease. *J Transl Med.* 2018;16(1):146.

25. Lin X, You Y, Wang J, Qin Y, Huang P, and Yang F. MicroRNA-155 deficiency promotes nephrin acetylation and attenuates renal damage in hyperglycemia-induced nephropathy. *Inflammation.* 2015;38(2):546-54.

26. Bijkerk R, Duijs JM, Khairoun M, Ter Horst CJ, van der Pol P, Mallat MJ, et al. Circulating microRNAs associate with diabetic nephropathy and systemic microvascular damage and normalize after simultaneous pancreas-kidney transplantation. *Am J Transplant.* 2015;15(4):1081-90.

27. Zhang J, Wu C, Dong J, Liu J, and Wei X. Downregulation of miR-181a alleviates renal fibrosis in diabetic nephropathy mice. *Int J Clin Exp Pathol.* 2018;11(8):4004-11.

28. Maity S, Bera A, Ghosh-Choudhury N, Das F, Kasinath BS, and Choudhury GG. microRNA-181a downregulates deptor for TGFbeta-induced glomerular mesangial cell hypertrophy and matrix protein expression. *Exp Cell Res.* 2018;364(1):5-15.

29. Wang LP, Geng JN, Sun B, Sun CB, Shi Y, and Yu XY. MiR-92b-3p is Induced by Advanced Glycation End Products and Involved in the Pathogenesis of Diabetic Nephropathy. *Evid Based Complement Alternat Med.* 2020;2020:6050874.

30. Cardenas-Gonzalez M, Srivastava A, Pavkovic M, Bijol V, Rennke HG, Stillman IE, et al. Identification, Confirmation, and Replication of Novel Urinary MicroRNA Biomarkers in Lupus Nephritis and Diabetic Nephropathy. *Clin Chem.* 2017;63(9):1515-26.

31. Ma J, Wang Y, Xu HT, Ren N, Zhao N, Wang BM, et al. MicroRNA: a novel biomarker and therapeutic target to combat autophagy in diabetic nephropathy. *Eur Rev Med Pharmacol Sci.* 2019;23(14):6257-63.

32. Liu F, Zhang ZP, Xin GD, Guo LH, Jiang Q, and Wang ZX. miR-192 prevents renal tubulointerstitial fibrosis in diabetic nephropathy by targeting Egr1. *Eur Rev Med Pharmacol Sci.* 2018;22(13):4252-60.

33. Ma X, Lu C, Lv C, Wu C, and Wang Q. The Expression of miR-192 and Its Significance in Diabetic Nephropathy Patients with Different Urine Albumin Creatinine Ratio. *J Diabetes Res.* 2016;2016:6789402.

34. Fiorentino L, Cavalera M, Mavilio M, Conserva F, Menghini R, Gesualdo L, et al. Regulation of TIMP3 in diabetic nephropathy: a role for microRNAs. *Acta Diabetol.* 2013;50(6):965-9.

35. Kolling M, Kaucsar T, Schauerte C, Hubner A, Dettling A, Park JK, et al. Therapeutic miR-21 Silencing Ameliorates Diabetic Kidney Disease in Mice. *Mol Ther.* 2017;25(1):165-80.

36. Wang X, Shen E, Wang Y, Li J, Cheng D, Chen Y, et al. Cross talk between miR-214 and PTEN attenuates glomerular hypertrophy under diabetic conditions. *Sci Rep.* 2016;6:31506.

37. Mu J, Pang Q, Guo YH, Chen JG, Zeng W, Huang YJ, et al. Functional implications of microRNA-215 in TGF-beta1-induced phenotypic transition of mesangial cells by targeting CTNNBIP1. *PLoS One.* 2013;8(3):e58622.

38. Wang J, Duan L, Tian L, Liu J, Wang S, Gao Y, et al. Serum miR-21 may be a Potential Diagnostic Biomarker for Diabetic Nephropathy. *Exp Clin Endocrinol Diabetes.* 2016;124(7):417-23.

39. El-Samahy MH, Adly AA, Elhenawy YI, Ismail EA, Pessar SA, Mowafy ME, et al. Urinary miRNA-377 and miRNA-216a as biomarkers of nephropathy and subclinical atherosclerotic risk in pediatric patients with type 1 diabetes. *J Diabetes Complications.* 2018;32(2):185-92.

40. Rudnicki M, Beckers A, Neuwirt H, and Vandesompele J. RNA expression signatures and posttranscriptional regulation in diabetic nephropathy. *Nephrol Dial Transplant.* 2015;30 Suppl 4:iv35-42.

41. Zhao B, Li H, Liu J, Han P, Zhang C, Bai H, et al. MicroRNA-23b Targets Ras GTPase-Activating Protein SH3 Domain-Binding Protein 2 to Alleviate Fibrosis and Albuminuria in Diabetic Nephropathy. *J Am Soc Nephrol.* 2016;27(9):2597-608.

42. Carney EF. Diabetic nephropathy: MiR-23b protects against fibrosis in diabetic nephropathy. *Nat Rev Nephrol.* 2016;12(4):197.

43. Liu H, Wang X, Liu S, Li H, Yuan X, Feng B, et al. Effects and mechanism of miR-23b on glucose-mediated epithelial-to-mesenchymal transition in diabetic nephropathy. *Int J Biochem Cell Biol.* 2016;70:149-60.

44. Liu Y, Li H, Liu J, Han P, Li X, Bai H, et al. Variations in MicroRNA-25 Expression Influence the Severity of Diabetic Kidney Disease. *J Am Soc Nephrol.* 2017;28(12):3627-38.

45. Koga K, Yokoi H Fau - Mori K, Mori K Fau - Kasahara M, Kasahara M Fau - Kuwabara T, Kuwabara T Fau - Imamaki H, Imamaki H Fau - Ishii A, et al. MicroRNA-26a inhibits TGF-beta-induced extracellular matrix protein expression in podocytes by targeting CTGF and is downregulated in diabetic nephropathy. *Diabetologia.* 2015;58(9):2169-80.

46. Wu L, Wang Q, Guo F, Ma X, Ji H, Liu F, et al. MicroRNA-27a Induces Mesangial Cell Injury by Targeting of PPARgamma, and its In Vivo Knockdown Prevents Progression of Diabetic Nephropathy. *Sci Rep.* 2016;6:26072.

47. Zhou Z, Wan J, Hou X, Geng J, Li X, and Bai X. MicroRNA-27a promotes podocyte injury via PPARgamma-mediated beta-catenin activation in diabetic nephropathy. *Cell Death Dis.* 2017;8(3):e2658.

48. Hou X, Tian J, Geng J, Li X, Tang X, Zhang J, et al. MicroRNA-27a promotes renal tubulointerstitial fibrosis via suppressing PPARgamma pathway in diabetic nephropathy. *Oncotarget.* 2016;7(30):47760-76.

49. Delic D, Eisele C, Schmid R, Baum P, Wiech F, Gerl M, et al. Urinary Exosomal miRNA Signature in Type II Diabetic Nephropathy Patients. *PLoS One.* 2016;11(3):e0150154.

50. Hsu YC, Chang PJ, Ho C, Huang YT, Shih YH, Wang CJ, et al. Protective effects of miR-29a on diabetic glomerular dysfunction by modulation of DKK1/Wnt/beta-catenin signaling. *Sci Rep.* 2016;6:30575.

51. Assmann TS, Recamonde-Mendoza M, Costa AR, Punales M, Tschiedel B, Canani LH, et al. Circulating miRNAs in diabetic kidney disease: case-control study and in silico analyses. *Acta Diabetol.* 2019;56(1):55-65.

52. Assmann TS, Recamonde-Mendoza M, de Souza BM, Bauer AC, and Crispim D. MicroRNAs and diabetic kidney disease: Systematic review and bioinformatic analysis. *Mol Cell Endocrinol.* 2018;477:90-102.

53. Pezzolesi MG, Satake E, McDonnell KP, Major M, Smiles AM, and Krolewski AS. Circulating TGF-beta1-Regulated miRNAs and the Risk of Rapid Progression to ESRD in Type 1 Diabetes. *Diabetes.* 2015;64(9):3285-93.

54. Guo J, Li J, Zhao J, Yang S, Wang L, Cheng G, et al. MiRNA-29c regulates the expression of inflammatory cytokines in diabetic nephropathy by targeting tristetraprolin. *Sci Rep.* 2017;7(1):2314.

55. Shao H, Huang Y, Hu HL, Fan WX, and Yin XN. Effect of miR-29c on renal fibrosis in diabetic rats via the AMPK/mTOR signaling pathway. *Eur Rev Med Pharmacol Sci.* 2019;23(14):6250-6.

56. Chien HY, Chen CY, Chiu YH, Lin YC, and Li WC. Differential microRNA Profiles Predict Diabetic Nephropathy Progression in Taiwan. *Int J Med Sci.* 2016;13(6):457-65.

57. Sole C, Cortes-Hernandez J, Felip ML, Vidal M, and Ordi-Ros J. miR-29c in urinary exosomes as predictor of early renal fibrosis in lupus nephritis. *Nephrol Dial Transplant.* 2015;30(9):1488-96.

58. Tang WB, Zheng L, Yan R, Yang J, Ning J, Peng L, et al. miR302a-3p May Modulate Renal Epithelial-Mesenchymal Transition in Diabetic Kidney Disease by Targeting ZEB1. *Nephron.* 2018;138(3):231-42.

59. Peng R, Zhou L, Zhou Y, Zhao Y, Li Q, Ni D, et al. MiR-30a Inhibits the Epithelial--Mesenchymal Transition of Podocytes through Downregulation of NFATc3. *Int J Mol Sci.* 2015;16(10):24032-47.

60. He M, Wang J, Yin Z, Zhao Y, Hou H, Fan J, et al. MiR-320a induces diabetic nephropathy via inhibiting MafB. *Aging (Albany NY).* 2019;11(10):3055-79.

61. Wang Q, Wang Y, Minto AW, Wang J, Shi Q, Li X, et al. MicroRNA-377 is up-regulated and can lead to increased fibronectin production in diabetic nephropathy. *FASEB J.* 2008;22(12):4126-35.

62. Wang L, and Li H. MiR-770-5p facilitates podocyte apoptosis and inflammation in diabetic nephropathy by targeting TIMP3. *Biosci Rep.* 2020.
