# Supplementary material for: Identification of Cooperative Gene Regulation Among Transcription Factors, LncRNAs, and MicroRNAs in Diabetic Nephropathy Progression
Source: Front Genet. 2020 Sep 1;11:1008. doi: 10.3389/fgene.2020.01008 (PMC7490338; doi:10.3389/fgene.2020.01008)
Supplement: Supplementary file 2 [file Table_2.DOCX]

**Table S2. Altered lncRNAs reported in human diabetic nephropathy studies**

| LncRNA | number of reports for upregulated | number of reports for downregulated | Targets | function | sample | Ref |
| --- | --- | --- | --- | --- | --- | --- |
| Blnc1 | 1 |  | NRF2/HO-1 and NF-κB pathways | Inflammation, oxidative stress and renal fibrosis | h, m | (1) |
| CACS2 |  | 2 |  | predict for the occurrence of chronic DN | h, m | (2, 3) |
| CDKN2B-AS1 | 1 |  | miR-424-5p, HMGA2 | Proliferation, ECM accumulation and PI3K/AKT signaling | h | (4) |
| GAS5 | 1 |  | BNIP3, FN1, miR-96-5p | Pro-apoptotic roles,regulates fibrosis and its mechanism in TGF-β1-treated renal proximal tubular cell. | h, m | (5) |
| H19 | 1 |  | miR-675 | vitamin D (VD) receptor | h | (6) |
| HOTAIR | 1 |  |  | bystander in DKD | h, m | (7) |
| LINC00462 | 1 |  | AKT | High Glucose-Induced Apoptosis | h | (8) |
| LINC01619 |  | 2 | miR-27a, FOXO1 | Oxidative stress and podocyte injuries | h, r | (9) |
| MALAT1 | 9 |  | ZEB2, Foxo1, SIRT1, SRSF1, SAA3, miR-23, IL-6, TNF-α, β-catenin | Transcriptional regulation in EMT, fibrosis, and many pathways by binding to protein and miRNA | h, m, r | (10-18) |
| MEG3 | 2 |  | TLR4 | progression of DN | m | (19, 20) |
| MIAT |  | 1 | Nrf2 | high glucose induced renal tubular epithelial injury | h, r | (21) |
| NEAT1 | 2 |  | miR-27b-3p, ZEB1 | EMT accumulation | h, r | (22, 23) |
| NR_033515 | 1 |  | P38, ASK1, Fibronectin, α-SMA, E-cadherin, Vimentin | promoted proliferation, and inhibited apoptosis of MMC cells and increased the expression levels of proliferation-related genes | h | (24) |
| NR_038323 |  | 1 | miR-324-3p/DUSP1/p38MAPK and ERK1/2 | renal fibrosis | h, r | (25) |
| PRINS | 1 |  | Smad7 | decreased the viability of mouse podocyte cells | h, m | (26) |
| PVT1 | 3 |  | FOXA1, TGF-β1, PAI-1 and fibronectin | ECM accumulation, apoptosis, and fibrosis | h, m | (27-29) |
| Rpph1 | 1 |  | Gal-3/Mek/Erk | inflammation and cell proliferation | m | (30) |
| SOX2OT |  | 1 | SIRT1 | induce autophagy | h | (31) |
| TCF7 | 1 |  | miR-200c | Endoplasmic reticulum | h | (32) |
| TUG1 |  | 6 | TIMP3, PI3K, Akt, miR-21, Ppargc1a, miR-377, TRAF5 | Transcriptional activation by protein and miRNA binding in proliferation and ECM accumulation, renal fibrosis | h, m, r | (33-38) |
| XIST | 1 |  | miR-217, microRNA-93-5p, CDKN1A | miRNA binding for apoptosis | h, m | (39) |

**Reference**

1. Feng X, Zhao J, Ding J, Shen X, Zhou J, and Xu Z. LncRNA Blnc1 expression and its effect on renal fibrosis in diabetic nephropathy. *Am J Transl Res.* 2019;11(9):5664-72.

2. Yang H, Kan QE, Su Y, and Man H. Long Non-Coding RNA CASC2 Improves Diabetic Nephropathy by Inhibiting JNK Pathway. *Exp Clin Endocrinol Diabetes.* 2019;127(8):533-7.

3. Wang L, Su N, Zhang Y, and Wang G. Clinical Significance of Serum lncRNA Cancer Susceptibility Candidate 2 (CASC2) for Chronic Renal Failure in Patients with Type 2 Diabetes. *Med Sci Monit.* 2018;24:6079-84.

4. Li Y, Zheng LL, Huang DG, Cao H, Gao YH, and Fan ZC. LNCRNA CDKN2B-AS1 regulates mesangial cell proliferation and extracellular matrix accumulation via miR-424-5p/HMGA2 axis. *Biomed Pharmacother.* 2020;121:109622.

5. Lv L, Li D, Tian F, Li X, Jing Z, and Yu X. Silence of lncRNA GAS5 alleviates high glucose toxicity to human renal tubular epithelial HK-2 cells through regulation of miR-27a. *Artif Cells Nanomed Biotechnol.* 2019;47(1):2205-12.

6. Fan W, Peng YA-OhooX, Liang Z, Yang Y, and Zhang J. A negative feedback loop of H19/miR-675/EGR1 is involved in diabetic nephropathy by downregulating the expression of the vitamin D receptor. *J Cell Physiol.* 2019;234(10):17505-13.

7. Majumder S, Hadden MJ, Thieme K, Batchu SN, Niveditha D, Chowdhury S, et al. Dysregulated expression but redundant function of the long non-coding RNA HOTAIR in diabetic kidney disease. *Diabetologia.* 2019;62(11):2129-42.

8. Wang R, Yan Y, and Li C. LINC00462 is involved in high glucose-induced apoptosis of renal tubular epithelial cells via AKT pathway. LID - 10.1002/cbin.11231 [doi]. *Cell Biol Int.* 2019.

9. Bai X, Geng J, Li X, Wan J, Liu J, Zhou Z, et al. Long Noncoding RNA LINC01619 Regulates MicroRNA-27a/Forkhead Box Protein O1 and Endoplasmic Reticulum Stress-Mediated Podocyte Injury in Diabetic Nephropathy. *Antioxid Redox Signal.* 2018;29(4):355-76.

10. Fawzy MS, Abu AlSel BT, Al Ageeli E, Al-Qahtani SA, Abdel-Daim MM, and Toraih EA. Long non-coding RNA MALAT1 and microRNA-499a expression profiles in diabetic ESRD patients undergoing dialysis: a preliminary cross-sectional analysis. *Arch Physiol Biochem.* 2020;126(2):172-82.

11. Liu B, Qiang L, Wang GD, Duan Q, and Liu J. LncRNA MALAT1 facilities high glucose induced endothelial to mesenchymal transition and fibrosis via targeting miR-145/ZEB2 axis. *Eur Rev Med Pharmacol Sci.* 2019;23(8):3478-86.

12. Shi S, Yang J, Fan W, Zhou Z, Chen G, and Zhang J. Effects of LncRNA MALAT1 on microangiopathy and diabetic kidney disease in diabetic rats by regulating ERK/MAPK signaling pathway. *Minerva Med.* 2019.

13. Wu D, Cheng YG, Huang X, Zhong MW, Liu SZ, and Hu SY. Downregulation of lncRNA MALAT1 contributes to renal functional improvement after duodenal-jejunal bypass in a diabetic rat model. *J Physiol Biochem.* 2018;74(3):431-9.

14. Hu M, Wang R, Li X, Fan M, Lin J, Zhen J, et al. LncRNA MALAT1 is dysregulated in diabetic nephropathy and involved in high glucose-induced podocyte injury via its interplay with beta-catenin. *J Cell Mol Med.* 2017;21(11):2732-47.

15. Liu JY, Yao J, Li XM, Song YC, Wang XQ, Li YJ, et al. Pathogenic role of lncRNA-MALAT1 in endothelial cell dysfunction in diabetes mellitus. *Cell Death Dis.* 2014;5:e1506.

16. Lelli A, Nolan KA, Santambrogio S, Goncalves AF, Schonenberger MJ, Guinot A, et al. Induction of long noncoding RNA MALAT1 in hypoxic mice. *Hypoxia (Auckl).* 2015;3:45-52.

17. Li X, Zeng L, Cao C, Lu C, Lian W, Han J, et al. Long noncoding RNA MALAT1 regulates renal tubular epithelial pyroptosis by modulated miR-23c targeting of ELAVL1 in diabetic nephropathy. *Exp Cell Res.* 2017;350(2):327-35.

18. Zhou L, Xu DY, Sha WG, Shen L, and Lu GY. Long non-coding RNA MALAT1 interacts with transcription factor Foxo1 to regulate SIRT1 transcription in high glucose-induced HK-2cells injury. *Biochem Biophys Res Commun.* 2018;503(2):849-55.

19. Li J, Jiang X, Duan L, and Wang W. Long non-coding RNA MEG3 impacts diabetic nephropathy progression through sponging miR-145. *Am J Transl Res.* 2019;11(10):6691-8.

20. Zha F, Qu X, Tang B, Li J, Wang Y, Zheng P, et al. Long non-coding RNA MEG3 promotes fibrosis and inflammatory response in diabetic nephropathy via miR-181a/Egr-1/TLR4 axis. *Aging (Albany NY).* 2019;11(11):3716-30.

21. Zhou L, Xu DY, Sha WG, Shen L, Lu GY, and Yin X. Long non-coding MIAT mediates high glucose-induced renal tubular epithelial injury. *Biochem Biophys Res Commun.* 2015;468(4):726-32.

22. Ma J, Zhao N, Du L, and Wang Y. Downregulation of lncRNA NEAT1 inhibits mouse mesangial cell proliferation, fibrosis, and inflammation but promotes apoptosis in diabetic nephropathy. *Int J Clin Exp Pathol.* 2019;12(4):1174-83.

23. Wang X, Xu Y, Zhu YC, Wang YK, Li J, Li XY, et al. LncRNA NEAT1 promotes extracellular matrix accumulation and epithelial-to-mesenchymal transition by targeting miR-27b-3p and ZEB1 in diabetic nephropathy. *J Cell Physiol.* 2019;234(8):12926-33.

24. Gao J, Wang W, Wang F, and Guo C. LncRNA-NR_033515 promotes proliferation, fibrogenesis and epithelial-to-mesenchymal transition by targeting miR-743b-5p in diabetic nephropathy. *Biomed Pharmacother.* 2018;106:543-52.

25. Ge Y, Wang J, Wu D, Zhou Y, Qiu S, Chen J, et al. lncRNA NR_038323 Suppresses Renal Fibrosis in Diabetic Nephropathy by Targeting the miR-324-3p/DUSP1 Axis. *Mol Ther Nucleic Acids.* 2019;17:741-53.

26. Jiao H, Xie D, and Qiao Y. LncRNA PRINS is involved in the development of nephropathy in patients with diabetes via interaction with Smad7. *Exp Ther Med.* 2019;17(4):3203-8.

27. Liu DW, Zhang JH, Liu FX, Wang XT, Pan SK, Jiang DK, et al. Silencing of long noncoding RNA PVT1 inhibits podocyte damage and apoptosis in diabetic nephropathy by upregulating FOXA1. *Exp Mol Med.* 2019;51(8):1-15.

28. Millis MP, Bowen D, Kingsley C, Watanabe RM, and Wolford JK. Variants in the plasmacytoma variant translocation gene (PVT1) are associated with end-stage renal disease attributed to type 1 diabetes. *Diabetes.* 2007;56(12):3027-32.

29. Alvarez ML, Khosroheidari M Fau - Eddy E, Eddy E Fau - Kiefer J, and Kiefer J. Role of microRNA 1207-5P and its host gene, the long non-coding RNA Pvt1, as mediators of extracellular matrix accumulation in the kidney: implications for diabetic nephropathy. *PLoS One.* 2013;8(10):e77468.

30. Zhang P, Sun Y, Peng R, Chen W, Fu X, Zhang L, et al. Long non-coding RNA Rpph1 promotes inflammation and proliferation of mesangial cells in diabetic nephropathy via an interaction with Gal-3. *Cell Death Dis.* 2019;10(7):526.

31. Zhang Y, Chang B, Zhang J, and Wu X. LncRNA SOX2OT alleviates the high glucose-induced podocytes injury through autophagy induction by the miR-9/SIRT1 axis. *Exp Mol Pathol.* 2019;110:104283.

32. Liu H, and Sun HL. LncRNA TCF7 triggered endoplasmic reticulum stress through a sponge action with miR-200c in patients with diabetic nephropathy. *Eur Rev Med Pharmacol Sci.* 2019;23(13):5912-22.

33. Wang F, Gao X, Zhang R, Zhao P, Sun Y, and Li C. LncRNA TUG1 ameliorates diabetic nephropathy by inhibiting miR-21 to promote TIMP3-expression. *Int J Clin Exp Pathol.* 2019;12(3):717-29.

34. Li SY, and Susztak K. The long noncoding RNA Tug1 connects metabolic changes with kidney disease in podocytes. *J Clin Invest.* 2016;126(11):4072-5.

35. Long J, Badal SS, Ye Z, Wang Y, Ayanga BA, Galvan DL, et al. Long noncoding RNA Tug1 regulates mitochondrial bioenergetics in diabetic nephropathy. *J Clin Invest.* 2016;126(11):4205-18.

36. Duan LJ, Ding M, Hou LJ, Cui YT, Li CJ, and Yu DM. Long noncoding RNA TUG1 alleviates extracellular matrix accumulation via mediating microRNA-377 targeting of PPARgamma in diabetic nephropathy. *Biochem Biophys Res Commun.* 2017;484(3):598-604.

37. Zang XJ, Li L, Du X, Yang B, and Mei CL. LncRNA TUG1 inhibits the proliferation and fibrosis of mesangial cells in diabetic nephropathy via inhibiting the PI3K/AKT pathway. *Eur Rev Med Pharmacol Sci.* 2019;23(17):7519-25.

38. Shi CH, Huang Y, Li WQ, and Chen RG. Influence of LncRNA UCA1 on glucose metabolism in rats with diabetic nephropathy through PI3K-Akt signaling pathway. *Eur Rev Med Pharmacol Sci.* 2019;23(22):10058-64.

39. Yang J, Shen Y, Yang X, Long Y, Chen S, Lin X, et al. Silencing of long noncoding RNA XIST protects against renal interstitial fibrosis in diabetic nephropathy via microRNA-93-5p-mediated inhibition of CDKN1A. *Am J Physiol Renal Physiol.* 2019;317(5):F1350-F8.
